# Transcriptional response to the host cell environment of a multidrug-resistant *Mycobacterium tuberculosis* clonal outbreak Beijing strain reveals its pathogenic features

**DOI:** 10.1038/s41598-021-82905-x

**Published:** 2021-02-04

**Authors:** Pakorn Aiewsakun, Pinidphon Prombutara, Tegar Adriansyah Putra Siregar, Thanida Laopanupong, Phongthon Kanjanasirirat, Tanawadee Khumpanied, Suparerk Borwornpinyo, Pirut Tong-Ngam, Alisa Tubsuwan, Prapaporn Srilohasin, Angkana Chaiprasert, Wuthiwat Ruangchai, Prasit Palittapongarnpim, Therdsak Prammananan, Brian C. VanderVen, Marisa Ponpuak

**Affiliations:** 1 Department of Microbiology, Faculty of Science, Mahidol University, Bangkok, Thailand; 2 Pornchai Matangkasombut Center for Microbial Genomics, Department of Microbiology, Faculty of Science, Mahidol University, Bangkok, Thailand; 3 Omics Sciences and Bioinformatics Center, Faculty of Science, Chulalongkorn University, Bangkok, Thailand; 4 Microbiome Research Unit for Probiotics in Food and Cosmetics, Faculty of Sciences, Chulalongkorn University, Bangkok, Thailand; 5 Excellent Center for Drug Discovery, Faculty of Science, Mahidol University, Bangkok, Thailand; 6 Department of Biotechnology, Faculty of Science, Mahidol University, Bangkok, Thailand; 7 Institute of Molecular Biosciences, Mahidol University, Nakhon Pathom, Thailand; 8 Drug-Resistance Tuberculosis Research Fund, Siriraj Foundation, Faculty of Medicine Siriraj Hospital, Mahidol University, Bangkok, Thailand; 9 Office of Research and Development, Faculty of Medicine Siriraj Hospital, Mahidol University, Bangkok, Thailand; 10 National Center for Genetic Engineering and Biotechnology, National Science and Technology Development Agency, Pratumthani, Thailand; 11 Department of Microbiology and Immunology, Cornell University, Ithaca, NY USA

**Keywords:** Microbiology, Diseases

## Abstract

Tuberculosis is a global public health problem with emergence of multidrug-resistant infections. Previous epidemiological studies of tuberculosis in Thailand have identified a clonal outbreak multidrug-resistant strain of *Mycobacterium tuberculosis* in the Kanchanaburi province, designated “MKR superspreader”, and this particular strain later was found to also spread to other regions. In this study, we elucidated its biology through RNA-Seq analyses and identified a set of genes involved in cholesterol degradation to be up-regulated in the MKR during the macrophage cell infection, but not in the H37Rv reference strain. We also found that the bacterium up-regulated genes associated with the ESX-1 secretion system during its intracellular growth phase, while the H37Rv did not. All results were confirmed by qRT-PCR. Moreover, we showed that compounds previously shown to inhibit the mycobacterial ESX-1 secretion system and cholesterol utilisation, and FDA-approved drugs known to interfere with the host cholesterol transportation were able to decrease the intracellular survival of the MKR when compared to the untreated control, while not that of the H37Rv. Altogether, our findings suggested that such pathways are important for the MKR’s intracellular growth, and potentially could be targets for the discovery of new drugs against this emerging multidrug-resistant strain of *M. tuberculosis*.

## Introduction

According to the World Health Organization report, tuberculosis (TB) causes 10 million new cases and 1.5 million deaths annually worldwide^1^. With the emergence and rapid spread of multidrug-resistant (MDR) and extensively drug-resistant (XDR) *M. tuberculosis* (MTB) strains, the disease situation has worsen, and posed a serious global treat to the TB control program in many countries^1^. In particular, there is an increasingly wide spread of the modern Beijing genotype, which is regarded as a highly successful lineage of MTB, found to be associated with multidrug resistance, and large outbreaks^2,3^. The reasons for its high transmissibility are currently unclear, but thought to stem from the bacterial hyper-virulent phenotypes. Previous studies showed that, when compared to other strains, the Beijing strains had a greater survival rate inside the host macrophage cell, a higher bacterial load, and a greater mortality rate in animal models, as well as caused heavy AFB smear-positive sputum in human patients^4–9^. However, the molecular mechanism and factors underlying to the greater ability of the Beijing genotype to survive in the host, and extensively spread in the community remain to be determined.

MTB spends most of its infection cycle in host macrophages^10^. Mycobacteria are transmitted through air when the active TB patients cough, releasing infectious droplet nuclei from their lungs. Once entering the airway of recipients, MTB is phagocytosed by alveolar macrophages^11^. The resulting inflammatory response then drives the formation of granuloma structure by recruiting other immune cells to the surrounding infected areas, and thus restricting the growth of the mycobacteria within the phagosomes of the host macrophages. It was estimated that 90% of immunocompetent patients were able to contain the bacteria within the macrophages inside the granuloma, and hence had a latent TB infection^12^. However, at later time in life, these individuals had a 10% chance that the bacteria would reactivate, and cause an active TB disease^13^. It was also noted that 10% of individuals with a compromised or weak immune system could develop an active TB disease upon the exposure of the bacteria. The precise mechanism underlying the reactivation and causing an active TB disease is unknown. However, it is believed that the process involves bacterial growth spurt inside the macrophage cell, resulting in macrophage necrotic death, and in turn releasing the mycobacteria from the host cell into the extracellular environment^14^. Extracellular bacteria in the compromised granuloma could then be transmitted to others during coughing^15^. Therefore, the ability to grow inside the host macrophages will dictate the number of intracellular mycobacteria, and in turn will determine the number of extracellular MTB contained within the infectious transmitting droplet nuclei. Based on this, it is reasonable to assume that the increased ability of the mycobacteria to grow inside the host macrophages might result in its increased ability for transmission.

Previous epidemiological surveys of TB in Thailand revealed a large outbreak of multidrug-resistant TB in the Kanchanaburi province^3,16^. Whole genome sequencing in combination with genotypic analyses showed that the strain causing the outbreak, designated “MKR superspreader”, is a member of the modern Beijing genotype sequence type 10 (ST10)^3^, which is highly prevalent in many countries including Thailand^17–19^. It was later found that this particular strain has also spread to other regions^20^. The extensive spread of this emerging multidrug-resistant MTB strain in the community suggested that the MKR superspreader may have special abilities to adapt and grow inside the human host that might not be found in other local strains. To elucidate the biology of the MKR superspreader and identify potential factors contributing to its growth inside the host macrophages, in this study, we quantified changes in the transcriptional response of the MKR superspreader upon entering the host cells. By comparing its transcription profiles against those of the reference MTB, H37Rv, we found that the MKR superspreader uniquely upregulated genes functioning in the cholesterol degradation pathway and ESX-1 secretion system during its growth inside the host macrophages, confirmed by qRT-PCR. We also showed that, small molecules previously shown to inhibit these pathways were able to significantly reduce the intracellular survival of the MKR when compared to the untreated control, while H37Rv’s intracellular survival was not affected by these compounds. Our results suggested that these pathways may play unique and crucial roles in the MKR intracellular survivability, and thus might be targets for the discovery of new drugs against this emerging multidrug-resistant *M. tuberculosis* strain.

## Results

### Gene expression quantification

To elucidate MKR’s biology and identify potential factors contributing to its growth inside host macrophages, genome-wide gene expression levels of the reference MTB strain H37Rv, and the MKR superspreader were quantified by RNA-Seq analyses under three biological conditions: (i) during the exponential growth phase in 7H9 media (log-phase), (ii) 30 min post macrophage infection (T = 0), and (iii) 4 h post macrophage infection (T = 4). The 4 h time point was chosen as we were interested in investigating the early response of the bacteria to the hostile host cell environment. The results are summarised in Table 1 and Supplementary Dataset S1.

**Table 1:** RNA-Seq analysis statistics.

Experiment		Rep	Total reads	High-quality reads^a^	“Non-human” reads^b^	Aligned reads					Unaligned reads^c^
						Genes^d^	Ambiguous^d^	Low alignment quality^d^	No feature^d^	Total aligned reads^c^	
H35Rv	Log phase	1	40,436,194	37,494,942 (92.72%)	N/A	35,595,491 (95.46%)	479,559 (1.29%)	1,077,625 (2.89%)	137,077 (0.37%)	37,289,752 (99.45%)	205,190 (00.55%)
		2	33,260,151	29,975,555 (90.12%)	N/A	28,417,810 (95.25%)	424,760 (1.42%)	881,165 (2.95%)	110,753 (0.37%)	29,834,488 (99.53%)	141,067 (00.47%)
		3	43,495,077	41,052,086 (94.38%)	N/A	38,715,994 (94.90%)	563,711 (1.38%)	1,391,300 (3.41%)	125,766 (0.31%)	40,796,771 (99.38%)	255,315 (00.62%)
	T = 0	1	69,384,126	39,788,385 (57.34%)	10,272,043 (25.81%)	5,668,715 (93.14%)	30,144 (0.50%)	360,156 (5.92%)	27,449 (0.45%)	6,086,464 (59.25%)	4,185,579 (40.75%)
		2	49,069,412	43,216,311 (88.54%)	11,533,518 (26.69%)	5,721,097 (91.23%)	33,815 (0.54%)	487,648 (7.78%)	28,216 (0.45%)	6,270,776 (54.37%)	5,262,742 (45.63%)
		3	61,793,448	44,645,773 (72.25%)	11,871,446 (26.59%)	7,903,746 (94.92%)	61,297 (0.74%)	324,871 (3.90%)	36,578 (0.44%)	8,326,492 (70.14%)	3,544,954 (29.86%)
	T = 4	1	51,499,003	45,381,707 (88.12%)	11,771,501 (27.44%)	6,795,028 (94.13%)	31,231 (0.43%)	356,495 (4.94%)	35,934 (0.50%)	7,218,688 (61.32%)	4,552,813 (38.68%)
		2	37,533,515	31,889,646 (84.96%)	9,166,172 (28.74%)	5,502,028 (94.17%)	27,182 (0.47%)	282,361 (4.83%)	31,280 (0.54%)	5,842,851 (63.74%)	3,323,321 (36.26%)
		3	49,834,123	35,893,654 (72.03%)	12,408,152 (34.56%)	7,383,233 (92.19%)	33,263 (0.42%)	551,458 (6.89%)	40,899 (0.51%)	8,008,853 (64.55%)	4,399,299 (35.45%)
MKR	Log phase	1	47,208,119	41,914,952 (88.79%)	N/A	39,736,298 (95.23%)	610,170 (1.46%)	1,194,226 (2.86%)	187,760 (0.45%)	41,728,454 (99.56%)	186,498 (00.44%)
		2	35,798,457	28,025,257 (78.28%)	N/A	26,981,068 (96.68%)	315,930 (1.13%)	495,136 (1.77%)	115,074 (0.41%)	27,907,208 (99.58%)	118,049 (00.42%)
		3	39,691,565	33,012,205 (83.17%)	N/A	31,828,612 (96.83%)	530,170 (1.61%)	401,351 (1.22%)	110,259 (0.34%)	32,870,392 (99.57%)	141,813 (00.43%)
	T = 0	1	59,984,975	47,217,791 (78.72%)	8,170,834 (17.31%)	3,099,773 (93.47%)	12,993 (0.39%)	185,047 (5.58%)	18,406 (0.56%)	3,316,219 (40.59%)	4,854,615 (59.41%)
		2	51,631,051	47,992,656 (92.95%)	6,447,278 (13.43%)	1,688,256 (93.86%)	7,241 (0.40%)	93,583 (5.20%)	9,526 (0.53%)	1,798,606 (27.90%)	4,648,672 (72.10%)
		3	49,822,302	45,605,000 (91.53%)	7,876,348 (17.27%)	2,845,379 (93.46%)	13,139 (0.43%)	170,620 (5.60%)	15,255 (0.50%)	3,044,393 (38.65%)	4,831,955 (61.35%)
	T = 4	1	53,928,086	46,705,940 (86.61%)	6,740,593 (14.43%)	1,993,218 (94.22%)	6,129 (0.29%)	103,377 (4.89%)	12,736 (0.60%)	2,115,460 (31.38%)	4,625,133 (68.62%)
		2	33,601,205	29,535,628 (87.90%)	5,426,088 (18.37%)	1,155,969 (91.32%)	3,664 (0.29%)	99,314 (7.85%)	6,863 (0.54%)	1,265,810 (23.33%)	4,160,278 (76.67%)
		3	38,471,145	27,503,371 (71.50%)	5,483,082 (19.94%)	1,618,957 (92.98%)	4,674 (0.27%)	107,999 (6.20%)	9,525 (0.55%)	1,741,155 (31.76%)	3,741,927 (68.24%)

^a^The percentages are with respect to the total read numbers.

^b^With respect to the high-quality reads.

^c^With respect to the “non-human” reads.

^d^With respect to the total aligned reads.

An average of 47 million short-reads of RNA sequences were generated from the six experiments, each with three biological replicates. After sequencing adapter removal and read quality control, high-quality reads (57.34–94.38% of the total reads) were mapped to the human reference genome to remove human-associated RNA sequences. The remaining reads were then mapped to the reference MTB genome with gene annotation (GCA_000195955.2).

From the log-phase conditions, almost all of the reads could be mapped to the bacterial genome (99.38–99.58%), ranging between 27.91–41.73 million reads. 94.90–96.83% of the mapped reads were mapped to the genic regions, ranging between 26.98–39.74 million reads. Regarding the T = 0 and T = 4 settings, ~ 3.32–5.26 million reads did not map to either the reference human genome or bacterial genome. We analysed RNA-Seq data derived from uninfected macrophage host cells at T = 0 and T = 4 as negative controls, and found a comparable level of unmapped reads, ranging between 4.81–6.04 million reads, suggesting that they were sequencing background noise. Under these experimental settings, 91.23–94.92% of the mapped reads were mapped to the MTB genic regions. In terms of absolute numbers, the number of reads mapped to the genic regions for the H37Rv samples were between 5.50–7.90 million reads, while those of the MKR samples were only between 1.12–3.10 million reads (Table 1). These were lower than the generally recommended number of 5 million reads per sample for an identification of differentially expressed genes in bacteria. Our analyses were thus likely conservatively biased—genes that were detected by our analyses were likely those with strong effects, high expression levels and/or with high degrees of differential expression levels among experimental conditions. For weakly expressed genes and metabolic pathways that deviated only slightly among conditions, RNA-Seq experiments with greater sequencing depths and/or more biological replicates might be required.

**Figure 1:**
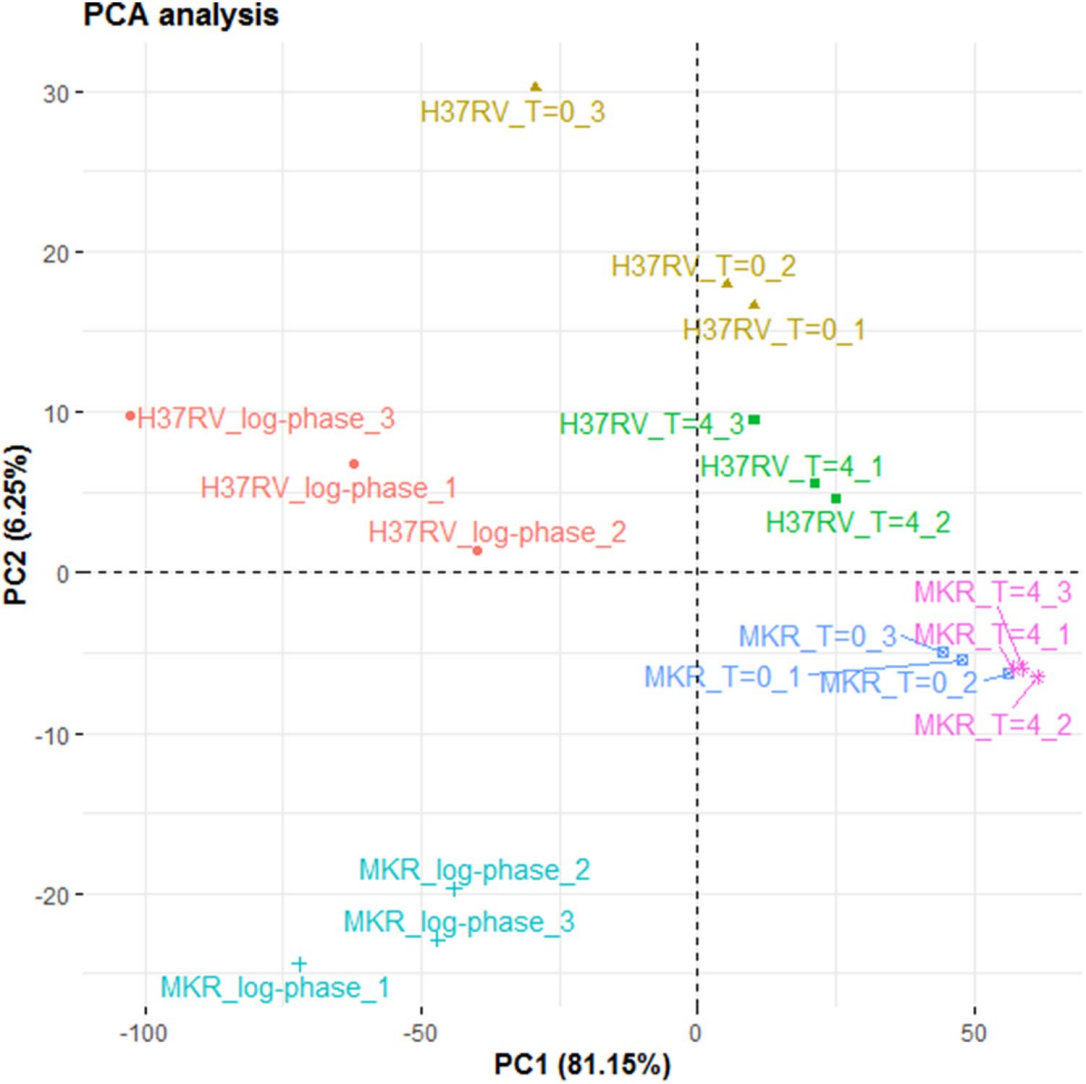
Principle component analysis. Samples from the same experimental conditions tend to cluster together according to their gene expression profiles. The first principle component (PC1) appears to capture the differences of the gene expression profiles across the three experimental conditions (log-phase, T = 0 and T = 4), and the second component appears to capture the differences of the gene expression profiles among H37RV and MKR superspreader.

Principle component analysis showed that the three biological replicates of each experiment tended to cluster together (Fig. 1), indicating that they had similar gene expression profiles. Interestingly, samples from the same experimental conditions appeared to separate along the first principle component axis, while those from the same MTB strain appeared to separate along the second principle component axis. These patterns suggested that there were some similarities between the gene expression profiles across different bacterial strains that were subjected to the same experimental conditions, but at the same time, there were also unique genes that were differentially, but consistently, expressed in the two bacteria regardless of the experimental conditions that they were subjected to.

### Differential gene expression and gene functional classification analyses

**Figure 2:**
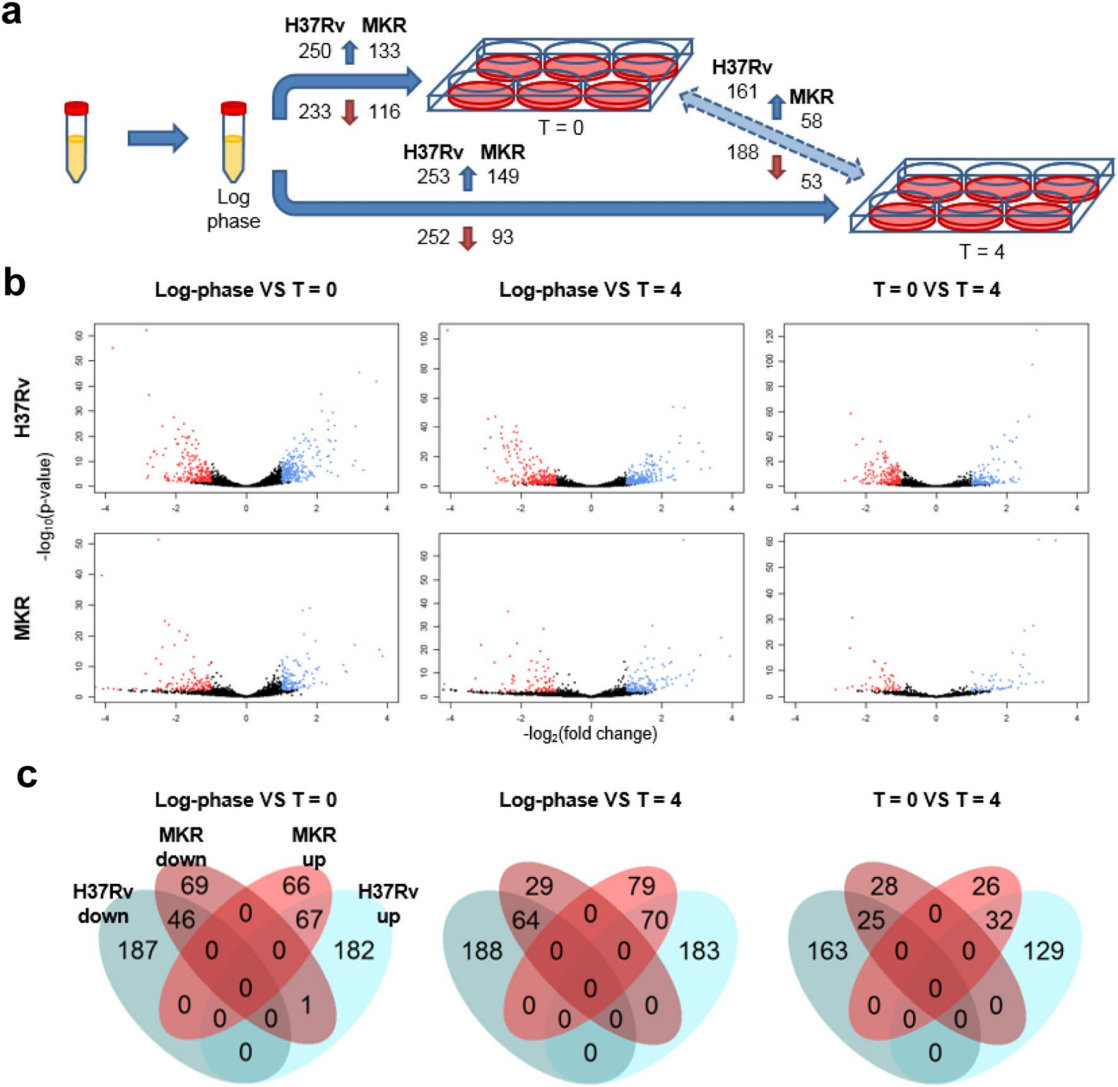
Differential gene expression analyses. (**a**) differentially expressed genes were determined among the three biological conditions (baseline VS experimental group: log-phase VS T = 0, log-phase VS T = 4, and T = 0 VS T = 4). The numbers of significantly up-regulated genes are shown on the top of the arrows while the numbers of significantly down-regulated genes are shown on the bottom. (**b**) Volcano plots (red: down-regulated genes; and blue: up-regulated genes). (**c**) Venn diagrams illustrating the numbers of differentially expressed genes common and unique among different strains of *M. tuberculosis*.

Differentially expressed genes were determined among the three biological settings (Fig. 2 and Supplementary Dataset S2). Genes with low expression levels were not included in the analyses. For each comparison pair, the baseline group was the one that came earlier in the experimental design (Fig. 2a), and directionality of the change (i.e. up-regulation or down-regulation) were with respect to the baseline group, and thus to time. For the log-phase VS T = 0 pair, the gene expression levels under the T = 0 condition were compared against those under the log-phase, and up-regulated genes were those whose expression levels were greater under the T = 0 condition, for example.

Overall, we found that the reference strain H37Rv had greater numbers of differentially expressed genes compared to the MKR strain, and consistently across all comparisons, about half of the differentially expressed genes in MKR were common with those of H37Rv (Fig. 2b,c). Considering common differentially expressed genes, all genes that were found to be up-regulated in H37Rv were also found to be up-regulated in MKR, and the same pattern was observed for down-regulated genes. The only exception was *Rv3742c*, which was found to be up-regulated in H37Rv, but down-regulated in MKR under the T = 0 condition compared to the log-phase condition. *Rv3742c* is a gene coding for an oxidoreductase protein of an unknown function previously found to be up-regulated in H37Rv after infection into human macrophage-differentiated THP-1 cells^21^.

**Figure 3:**
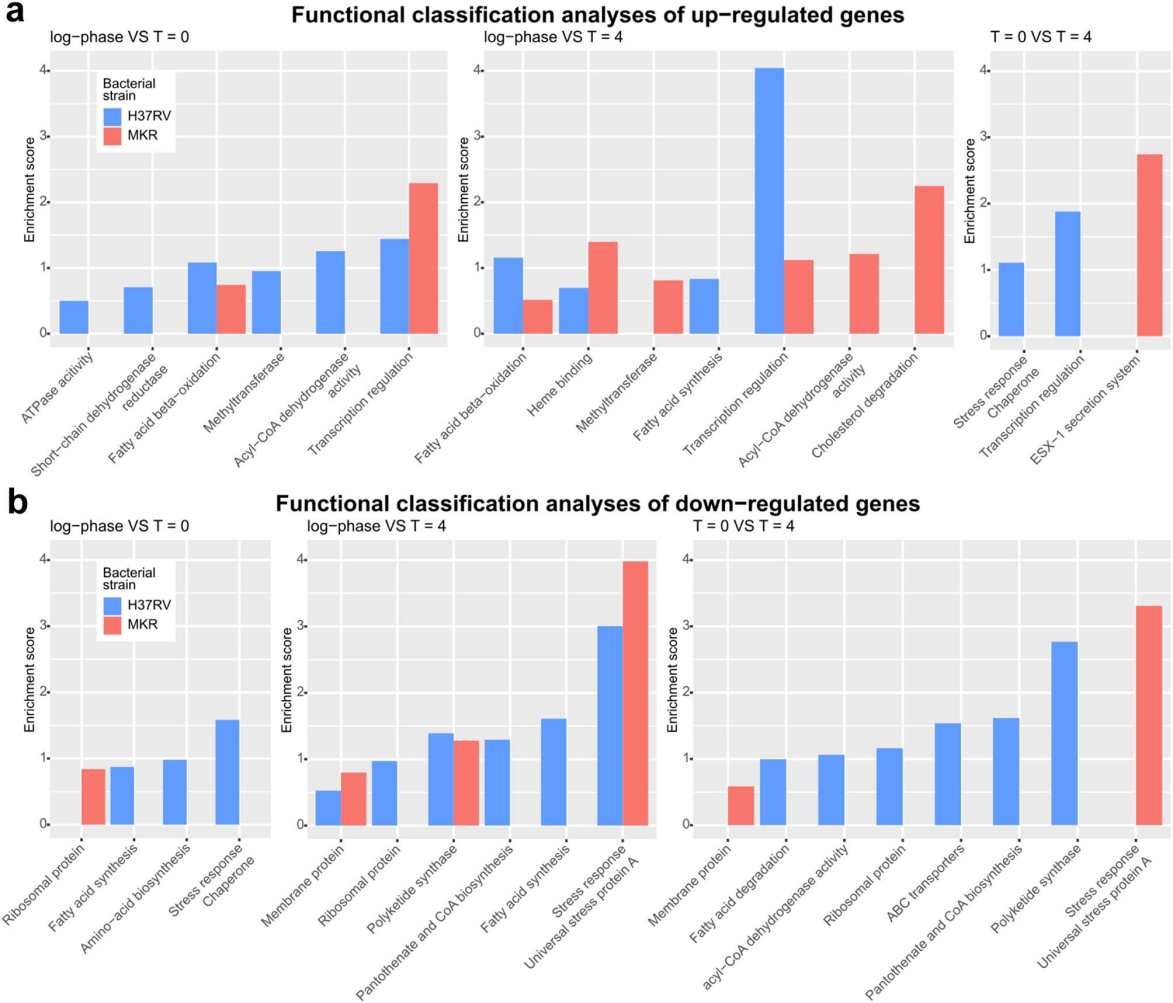
Gene functional classification analyses. (**a**) up-regulated genes were annotated with various functional terms, and gene functional classification analyses were performed by using DAVID Bioinformatics Resources 6.8 under the default settings. Baseline VS experimental group: log-phase VS T = 0 (left), log-phase VS T = 4 (middle), and T = 0 VS T = 4 (right). (**b**) down-regulated genes were annotated with functional terms and gene functional classification analyses were performed as in **(a)**.

Next, we performed in silico gene functional classification analyses on the differentially expressed genes. Our analyses showed that H37Rv and MKR had different sets of differentially expressed genes, and the profiles varied across experimental condition comparisons (Fig. 3a,b and Supplementary Dataset S3). We found that there were more genes functioning in the stress response down-regulated in the intracellular MKR superspreader compared to that of H37Rv, and more interestingly, we found that the cholesterol degradation pathway and the ESX-1 virulence molecule secretion system appeared to be up-regulated overall in the MRK superspreader after the host cell infection, but not in H37Rv.

### Cholesterol degradation pathway is important for intracellular survivability of MKR

Previous studies showed that some MTB strains preferentially metabolise cholesterol during the growth inside the host cell^14,22^. Recent evidence also demonstrated the importance of this metabolic pathway in virulence and pathogenesis of mycobacteria^23–27^. Cholesterol was found to be utilised by MTB for not only the energy production and gluconeogenesis, but also for the biosynthesis of lipid virulent factors, such as phthiocerol-dimycocerosate (PDIM) and sulfolipids (SLs)^28^.

**Figure 4:**
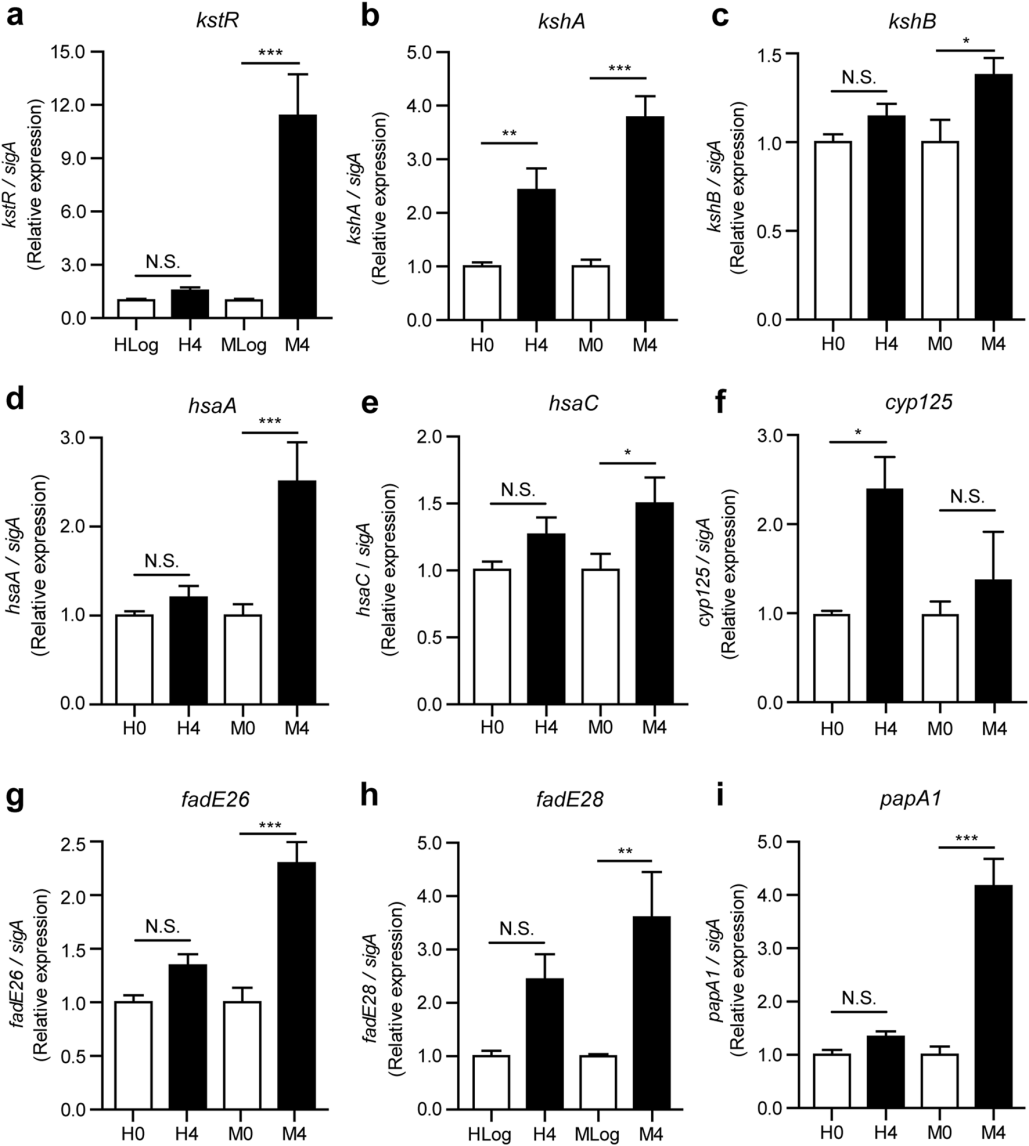
Expression levels of genes in the cholesterol degradation pathway. (**a–i**) THP-1 macrophages were infected with the *M. tuberculosis* reference strain H37Rv (H) or the MKR superspreader (M) for 4 h. The expression levels of various genes in the cholesterol degradation pathway were quantified by qRT-PCR. 2^-∆∆ct^ is used for normalisation and relative quantification. One-way ANOVA with Tukey's multiple comparison test was used to determine which pairs of expression levels were significantly different (*: p < 0.05; **: p < 0.01; and ***: p < 0.001).

qRT-PCR analyses showed that, indeed, there were many genes functioning in the cholesterol degradation pathway up-regulated in the MKR strain during its intracellular growth, and only a few were found to be up-regulated in the H37Rv strain (Fig. 4), in agreement with our RNA-seq analyses (Fig. 3 and Supplementary Dataset S3). Investigated genes included *kstR* (Rv3574) (Fig. 4a)—a recently identified transcriptional regulator, controlling the expression of genes involved in cholesterol degradation, lipid import, and production of virulence molecules^29,30^. Other genes included *kshA* (Rv3526), *kshB* (Rv3571), *hsaA* (Rv3570c), and *hsaC* (Rv3568c) (Fig. 4b–e), which encode proteins that digest RingsA/B, and *cyp125* (Rv3545c), *fadE26* (Rv3504), and *fadE28* (Rv3544c) (Fig. 4f–h), which encode proteins that degrade side chains of cholesterol molecules^30^. Furthermore, the expression of *papA1* (Rv3824c) (Fig. 4i), which encodes a key enzyme involved in sulfolipid-1 (SL-1) biosynthesis^31^, was also found to be significantly up-regulated in the intracellular MKR superspreader, but not in H37Rv.

**Figure 5:**
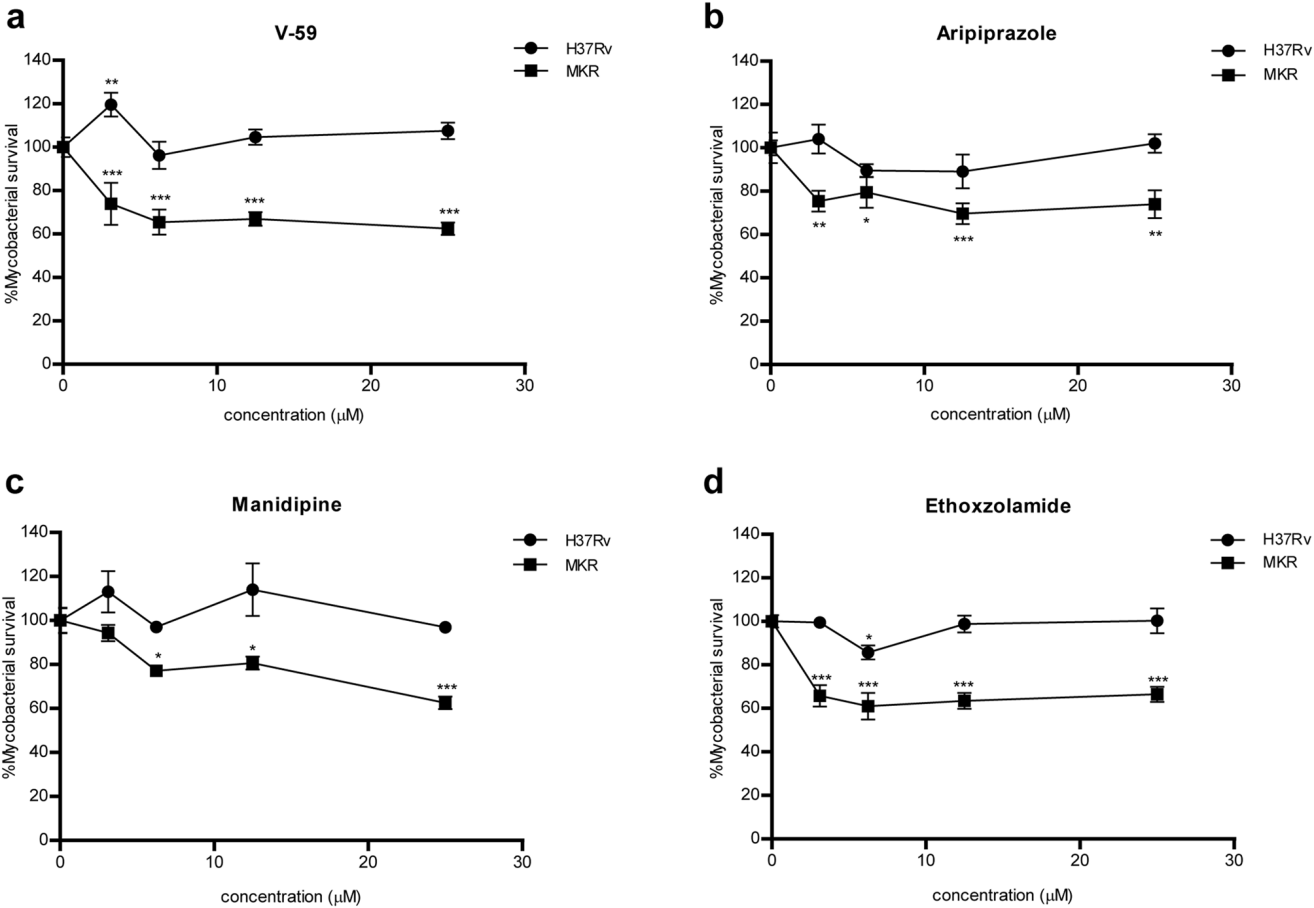
Intracellular survival odds of the MKR superspreader and H37Rv upon the treatments of V-59, aripiprazole, manidipine and ethoxzolamide. (**a–d**) THP-1 cells were infected with mCherry-expressing MKR superspreader or H37Rv for 30 min, and were treated with media containing various concentrations of (**a**) V-59, (**b**) aripiprazole, (**c**) manidipine and (**d**) ethoxzolamide for 3 days. Cells were then fixed and processed for high-content image analysis to quantify the number of mycobacteria per host cell. Percent mycobacterial survival at each concentration was calculated relative to that of the non-treated control group set to 100%. Two-way ANOVA with Bonferroni post-tests were used to determine if the intracellular survival levels were significantly different from that of the control group (*: p < 0.05; **: p < 0.01; and ***: p < 0.001).

To determine if cholesterol degradation is important for the intracellular growth of MKR, we infected human macrophage-differentiated THP-1 cells with the MKR superspreader and H37Rv, and treated the cells with an MTB cholesterol utilisation inhibitor, V-59, a derivative of the orphan cholesterol utilization inhibitor V-12-007958^32^. The experiment showed that V-59 treatment significantly reduced the survival of MKR when compared to the untreated control, but not of H37Rv (Fig. 5a). Mirroring this observation, treatment of MTB-infected macrophages with aripiprazole and manidipine—FDA-approved drugs known to alter cholesterol transportation of the host cells^33^—also resulted in a significant decrease of MKR's intracellular viability when compared to the untreated control, but not that of H37Rv (Fig. 5b,c). These findings suggested a crucial and unique role of the cholesterol degradation pathway in the intracellular growth of the MKR.

To further investigate the importance of cholesterol degradation, we knocked down the gene *kstR* (Rv3574), a transcriptional regulator of genes involved in cholesterol degradation, using the CRISPRi system^34^. Upon the knockdown induction by ATc addition, around 50% knockdown efficiency was achieved in the treated MKR and H37Rv groups, as determined by qRT-PCR (Supplementary Figure S1). We found that KstR-deficient MKR had a markedly lower intracellular survivability inside the host THP-1 cells compared to the uninduced control. This result further supported an important role of this gene in MKR’s intracellular growth. The same pattern was also observed for H37Rv however, but to a lesser extent (Supplementary Figure S1). Given that chemical compounds interfering with the bacterial cholesterol utilisation and host cholesterol transportation did not decrease the intracellular survival of H37Rv (Fig. 5a–c), the observed decrease in the intracellular survival of H37Rv upon the *kstR* knockdown could be due to other roles of this gene, for example, in lipid import and virulence molecule production as mentioned above.

### ESX-1 secretion system of virulence molecules is important for intracellular survivability of MKR

**Figure 6:**
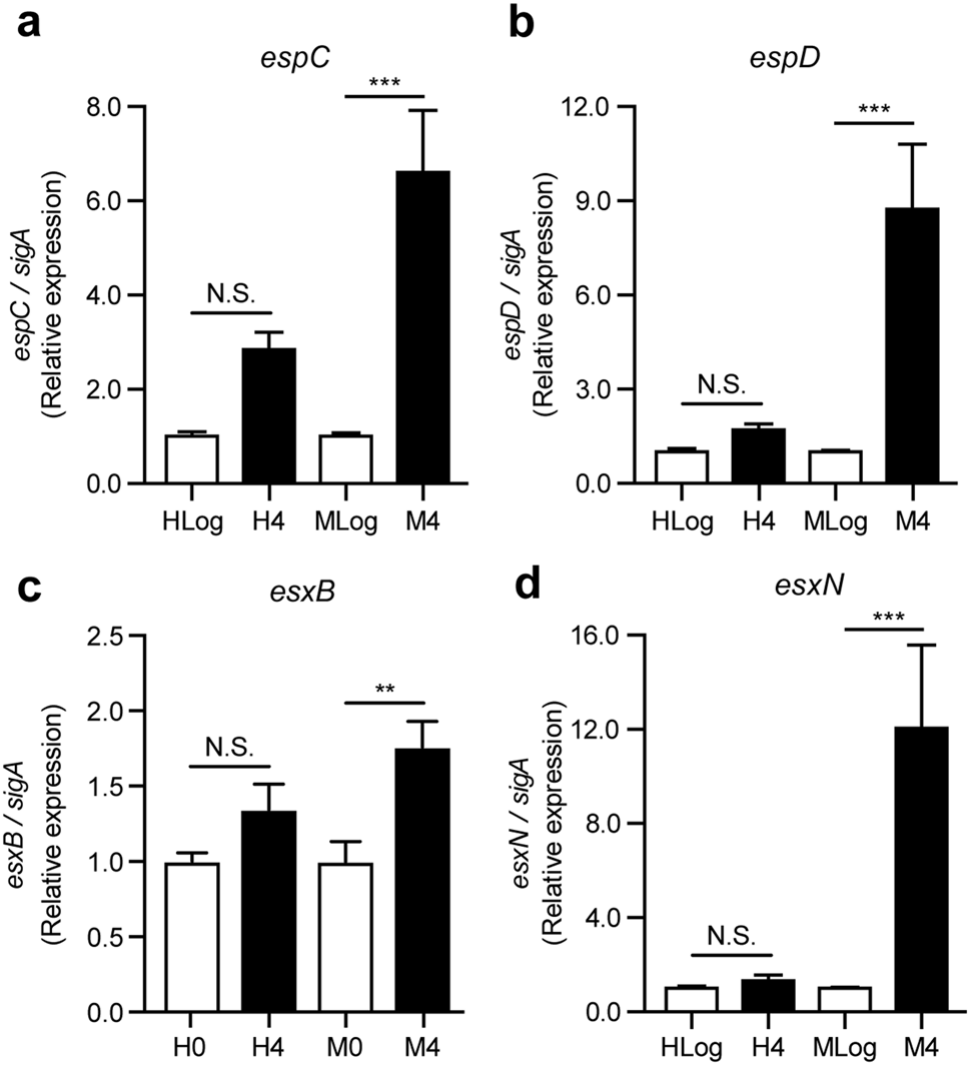
Expression levels of genes involved in the ESX-1 secretion system: *espC*, *espD*, *esxB*, and *esxN*. (**a–d**) THP-1 macrophages were infected with the *M. tuberculosis* reference strain H37Rv (H) or the MKR superspreader (M) for 4 h. The expression levels of various genes involved in the ESX-1 secretion system were quantified by qRT-PCR. 2^-∆∆ct^ is used for normalisation and relative quantification. One-way ANOVA with Tukey's multiple comparison test was used to determine which pairs of expression levels were significantly different (*: p < 0.05; **: p < 0.01; and ***: p < 0.001).

MTB uses the ESX-1 system to secrete virulence proteins into the host cell, which leads to phagosomal membrane disruption, and mycobacterial escape into the host cytosol, promoting the growth of the bacteria, and subsequently inducing host cell necrosis^35^. Our RNA-Seq analyses revealed that the ESX-1 secretion system was uniquely up-regulated in the MKR superspreader, but not in H37Rv, during its intracellular growth (Fig. 3). qRT-PCR analyses of four genes involved in the ESX-1 system, namely *espC* (Rv3615c), *espD* (Rv3614c), *esxB* (Rv3874), and *esxN* (Rv1793), showed that their expressions were indeed significantly increased in the MKR superspreader upon entering the host cell, and not in H37Rv (Fig. 6a–d), consistent with the RNA-Seq results.

To further investigate the importance of this pathway, human macrophage-differentiated THP-1 cells were infected with the MKR superspreader and H37Rv as described above, and were treated with ethoxzolamide—an FDA-approved drug known to inhibit the ESX-1 secretion system^36^. Again, we observed a significant decrease in the MKR's intracellular survivability upon the treatment of ethoxzolamide compared to the untreated control, but not in H37Rv (Fig. 5d). These findings suggested a crucial role of the ESX-1 secretion system in the intracellular growth of the MKR superspreader.

We also knocked down one of the genes in this pathway, *espD*, using the CRISPRi system^34^. Upon the knockdown induction by ATc addition, the expression level of the gene *espD* was determined to be decreased by ~ 25% by qRT-PCR both in the treated MKR and H37Rv (Supplementary Figure S1). These EspD-deficient mycobacteria had lower intracellular viabilities compared to the uninduced control groups, although the reduction was lower in H37Rv (Supplementary Figure S1). This result supported the vital role of EspD in MKR’s intracellular growth. Given that the inhibitor of ESX-1 secretion system did not affect the intracellular survival of H37Rv (Fig. 5d), and in fact the protein EspD was also found to be secreted independently of the ESX-1 secretion system^37^, the decrease in the intracellular survival of H37Rv upon the *espD* knockdown may suggest an unknown function of this gene in other pathways.

### Effects of V-59, aripiprazole, manidipine and ethoxzolamide on other MTB clinical isolates

**Figure 7:**
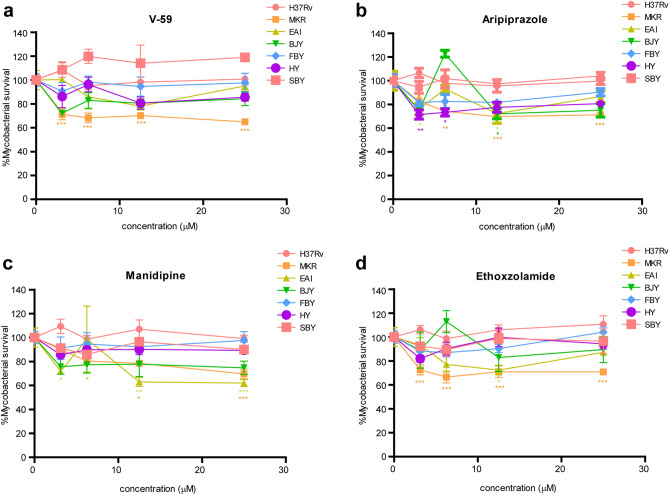
Intracellular survival odds of the MKR, H37Rv, and different clinical isolates upon the treatments of V-59, aripiprazole, manidipine and ethoxzolamide. (**a–d**) THP-1 cells were infected with Alexa-568-labelled MKR, H37Rv, or different clinical isolates for 30 min, and were treated with media containing various concentrations of (**a**) V-59, (**b**) aripiprazole, (**c**) manidipine and (**d**) ethoxzolamide for 3 days. Cells were then fixed and processed for high-content image analysis to quantify the number of mycobacteria per host cell. Percent mycobacterial survival at each concentration was calculated relative to that of the non-treated control group set to 100%. Two-way ANOVA with Bonferroni post-tests were used to determine if the intracellular survival levels were significantly different from that of the control group (*: p < 0.05; **: p < 0.01; and ***: p < 0.001).

As we observed that the intracellular survival of the MKR strain was markedly decreased upon the treatments of V-59, aripiprazole, manidipine and ethoxzolamide, while that of the laboratory reference strain H37Rv was not (Fig. 5), we then further tested these compounds on other Thai clinical isolates, namely EAI, BJY, FBY, HY, and SBY^38^. The intracellular viability of the MKR was again observed to significantly reduce upon the treatments of the cholesterol utilisation inhibitor V-59 and the ESX-1 secretion system inhibitor ethoxzolamide when compared to the untreated control, but such effect was not observed in other clinical isolates (Fig. 7a,d). The treatment of the host cholesterol transport inhibitors aripiprazole and manidipine also markedly decreased the intracellular growth of the MKR when compared to the untreated control, but unlike ethoxzolamide and V-59, these compounds were also able to reduce the intracellular survival of the EAI, BJY, and HY strains at some concentrations (aripiprazole at 3.125 μM for the HY and EAI strains, 6.25 μM for the HY strain, and 12.5 μM for EAI and BJY strains; manidipine at 3.125, 12.5, and 25 μM for the EAI strain) (Fig. 7b,c).

We also determined the expression levels of *hsaA*, *papA1*, and *espD* genes in these clinical isolates during their growth inside THP-1 cells by qRT-PCR (Supplementary Figure S2). These genes were selected as they play crucial roles in cholesterol degradation, SL1 synthesis, and ESX-1 secretion system, respectively^31,37,39^, and all were found to be up-regulated in the MKR superspreader (Figs. 4 and 6). Our experiments showed that, unlike the MKR, none of the other clinical isolates examined upregulated these three genes simultaneously; we found that while the BJY strain significantly upregulated the *hsaA* gene*,* and the SBY strain significantly upregulated the *papA1* gene, none upregulated the *espD* gene upon the host cell infection. Altogether, our results confirmed that cholesterol degradation and ESX-1 secretion pathways are important for the intracellular growth of the MKR superspreader.

## Discussion

Multidrug-resistant tuberculosis is a serious threat to the TB treatment and prevention programs around the world. Previous epidemiological studies have identified a large outbreak of MDR-TB in Kanchanaburi, Thailand, and the causative agent was determined to be a member of MTB Beijing ST10 genotype^3,16^. This emerging multidrug-resistant MTB strain was named “MKR superspreader”, and later was found to also spread to other regions^20^. However, the mechanism underlying its extensive spread in the community remained unclear. In this study, we attempted to elucidate the MKR’s biology by using RNA-Seq analyses to determine genetic factors involved in its intracellular growth. Our transcriptome analyses determined several genes to be up- and down-regulated in the MKR superspreader upon entering the host macrophages (Fig. 2).

Functional classification analyses of differentially expressed genes revealed that genes involved in cholesterol degradation and the ESX-1 secretion system were uniquely up-regulated in MKR (and not in H37Rv) after entering the host cell (Fig. 3), confirmed by qRT-PCR (Figs. 4 and 6). Restriction of the intracellular growth of MKR by V-59, and ethoxzolamide, both of which were previously shown to interfere with the aforementioned pathways^32,36^, corroborated the importance of these pathways in the intracellular growth of the bacterium (Figs. 5 and 7). In addition, through similar experiments, we identified two FDA-approved drugs, aripiprazole and manidipine, previously shown to interfere with the host cholesterol transportation for their new anti-mycobacterial activity (Figs. 5 and 7).

Cholesterol is a preferred substrate for energy production and glucogenesis by MTB^14^. Recent studies also showed the codependency of cholesterol and fatty acid metabolism for the synthesis of virulence lipids^28^. Cholesterol degradation results in the production of excess propionyl-CoA, a compound required for biosynthesis of various virulence lipids, including PDIM, and SLs^40^. PDIM was shown to be important for the MTB resistance to the host immune mediators^41–43^, and the escape of the bacteria from macrophage phagosome^44^, resulting in the bacterial intracellular growth spurt, and subsequently the necrotic death of the host cells^14^. SLs were shown to inhibit the production of TNF-α, a host cytokine important for granuloma formation, and hence the containment of MTB^45^, as well as inhibition of phagolysosome biogenesis in the host macrophages^46^. Interestingly, a recent work showed that SL-1, a member of SLs, is responsible for inducing coughing in MTB-infected guinea pigs^47^. As coughing facilitates MTB transmission, it might be possible that, by up-regulating the cholesterol degradation pathway, the MKR superspreader could increase the SL-1 production to induce coughing and thus enhance its transmission. Our analyses indeed identified *papA1* gene, which encodes a key enzyme in the SL-1 biosynthesis, to be up-regulated in the intracellular MKR (Supplementary Dataset S2 and Fig. 4), supporting this hypothesis.

In addition, a highly conserved set of KstR transcriptional regulators, including KstR and KstR1, were recently identified to control the expression of genes involved in cholesterol degradation, lipid import, and production of virulence molecules. Several genes in the KstR/KstR1 regulon were shown to be essential for MTB growth inside the mouse and macrophage models^29,30^. Our experiments identified several KstR-regulated genes to be up-regulated in MKR once it enters the host cell (Supplementary Dataset S2 and Fig. 4), and showed the decrease of the MKR’s intracellular survivability upon the treatment of V-59—an MTB cholesterol utilisation inhibitor. All of these observations are consistent with that the MKR superspreader up-regulates genes involved in cholesterol degradation, which promotes its intracellular growth and may increase the production of virulence lipids such as SL-1 to facilitate its transmission.

Based on our observations, we hypothesised that chemical compounds that alter host cholesterol transportation should also restrict the MKR’s intracellular growth. A recent work identified aripiprazole and manidipine, among other FDA-approved drugs, for their ability to alter cholesterol distribution in the THP-1 cells^33^. Our results showed that, indeed, these drugs could restrict the MKR intracellular viability (Figs. 5 and 7). Aripiprazole is currently available as a generic medication for treatment of several mental illnesses, such as depression and bipolar disorders^48^. Manidipine is a calcium channel blocker used to treat hypertension^49^. As these drugs are already available, and have been clinically approved, repurposing these drugs for the treatment of MDR-TB, especially those caused by the MKR superspreader, is an attractive option. These results warranted in vivo studies to further investigate the efficacy of these compounds in treating the MDR-TB. Besides their ability to restrict the MKR’s intracellular growth, these drugs were also found to be able to reduce the intracellular viability of some of the five additional Thai clinical isolates tested in this study, namely EAI, BJY, and HY, albeit at only some concentrations (Fig. 7). These results warranted further investigation on additional modes of action of these drugs, as the gene coding for a key enzyme in cholesterol degradation, HsaA, was not found to be upregulated in some of these strains (Supplementary Figure S2).

Besides the cholesterol degradation pathway, our RNA-Seq analyses identified several genes related to the ESX-1 secretion system to be up-regulated in the MKR strain after entering into the host macrophages. MTB ESX-1 system is well-known for its role in secretion of virulence molecules which disrupt the phagosomal membrane, leading to the escape of MTB into the cytosol^35^. The ESX-1 system is also required for the secretion of Esp proteins, such as EspA, EspB, and EspC^50^. EspB was previously shown to play roles in host cell cytotoxicity^51^, and EspA, and EspC, together with EspD, are required for ESX-1-mediated secretion^52^. Our data showed that the MKR viability inside the host cell decreased upon the treatment of an FDA-approved drug ethoxzolamide, an inhibitor of the ESX-1 secretion system^36^. Ethoxzolamide is currently used for the treatment of glaucoma and as diuretic. These results warranted further investigation on the possibility of repurposing this drug for the treatment of MDR-TB caused by the MKR strain.

## Materials and methods

### Cell and bacterial culture

Human monocytic THP-1 cells (ATCC TIB-202) were maintained in RPMI-1640 (Invitrogen), 10% FBS (Invitrogen), and 4 mM l-glutamine (Invitrogen), 10 mM HEPES (Invitrogen), 1 mM sodium pyruvate (Invitrogen), 4.5 g/L glucose (Invitrogen) and 0.05 mM 2-mercaptoethanol (Invitrogen). Cells were cultured at 37 °C and 5% CO_2_. *M. tuberculosis* reference strain H37Rv (BEI Resources; ATCC 25618), *M. tuberculosis* Beijing strain MKR superspreader (DS 5538)^3,16^, and *M. tuberculosis* Thai clinical isolates^38^ (FBY, BJY, EAI, SBY, and HY) were cultured in Middlebrook 7H9 broth supplemented with 0.05% Tween 80, 0.2% glycerol, and 10% oleic acid, albumin, dextrose, and catalase (BD Biosciences) at 37 °C and homogenised to generate single-cell suspension.

### Macrophage infection and total RNA extraction

To differentiate human monocytic THP-1 cells into macrophages, the cells were treated with 100 nM PMA (Sigma) for 24 h in 6-well plates (1.5 × 10^6^ cell per well). The cells were then washed with complete media three times, and rested in complete media for 24 h. Bacterial infection was carried out by co-culturing the bacteria with macrophages for 30 min with MOI of 20 as previously described^53^. The infected cells were washed three times with complete media to remove uninternalised mycobacteria (T = 0) and were further incubated with complete media for 4 h (T = 4). At T = 0 and T = 4, the media were removed, and Trizol (ThermoFisher) was added to solubilise the infected cells. In addition, uninfected cells (T = 0 and T = 4) and log-phase mycobacteria (OD = 1.0; grown in 7H9 media) were used as controls, and were also solubilised in Trizol. The total nucleic acids were released from the host and mycobacteria cells by bead-beating with 0.1 mm zirconia beads for 3 min (1 min bead-beat and 1 min rest on ice). Samples were centrifuged at 13,000 rpm for 3 min and supernatants were transferred into DNase RNase-free microcentrifuge tubes. Nucleic acids were isolated from each sample by the chloroform extraction followed by isopropanol precipitation method^54^. DNase (ThermoFisher) was added to the precipitated nucleic acids to degrade the genomic DNAs. Total RNAs were isolated by using the RNeasy kit (QIAGEN) according to the manufacturer’s instruction.

### RNA-Seq library construction and sequencing

The concentration of the total RNAs was measured by using DeNovix fluorometer (DeNovix). Sample purity was checked by using Nanodrop (Thermofisher). Integrity of the total RNAs was assessed by using Agilent 2100 Bioanalyzer (Agilent). Approximately 1 μg of the total RNAs from each sample was used to create individually indexed strand-specific RNA-Seq libraries by using QIAseq FastSelect RNA removal and QIAseq stranded total RNA library preparation kits (QIAGEN). Eukaryotic and prokaryotic rRNAs were removed by adding 1 μL of QIAseq FastSelect –rRNA HMR (Human, mouse, rat) and QIAseq FastSelect –5S/16S/23S-rRNA (bacteria) removal solution (QIAGEN, USA), and the reactions were subjected to fragmentation and cDNA synthesis. AMPure XP beads (Beckman Coulter Genomic) were used to separate the cDNAs from the reaction mix. Indexing adapters were ligated to the cDNAs, and subsequently all cDNA libraries were inspected for quality by using Agilent 2100 Bioanalyzer (Agilent) and quantified with DeNovix fluorometer (DeNovix). The indexed sequencing libraries were pooled in equimolar quantity and subjected to cluster generation and paired-end 2 × 150 nucleotide read sequencing on Illumina Hiseq sequencer.

### Quantifying the number of reads per transcript

To trim the adapters and to remove low-quality reads, the total RNA sequences from each experiment were pre-processed by Trimmomatic V0.32^55^ with the following parameters, ILLUMINACLIP: TruSeq3-PE.fa:2:30:10, LEADING:3, TRAILING:3, SLIDINGWINDOW:4:15, and MINLEN:36. The remaining high-quality reads were mapped to the prebuilt index of the human reference genome GRCh38 by using HISAT2^56^ to remove human associated RNA sequences under the default settings. The pair-end reads that were not mapped to the human genome were mapped to the MTB H37Rv reference genome (GCA_000195955.2) by using bowtie 2 V2.3.5.1^57^ under the ‘end-to-end’ mode. To quantify the number of reads per transcript, the read alignments were processed by *htseq-count*, implemented in HTSeq V0.11.2^58^. The counting ignored secondary alignments, supplementary alignments, and reads with alignment quality lower than 10. The corresponding gene annotations of the *M. tuberculosis* H37Rv reference genome (GCA_000195955.2) was obtained from http://bacteria.ensembl.org.

Principle component analysis was performed to examine if samples from the same experimental conditions have similar gene expression profiles or not. Genes with low expression levels, i.e. those with a mean value of read-mapping less than ten across all 6 experiments × 3 replicates = 18 datasets, were excluded from the analysis to reduce the noise. This filter removed 810 out of 4109 genes from the dataset, but in terms of read-mapping, they only accounted for 0.06% of the total read numbers at the maximum, suggesting that those genes were indeed not significantly expressed. Gene counts were scaled to have unit variance and zero mean prior the analysis. The analysis was performed in R, using *prcomp* function.

### Differential gene expression analyses

DESeq2 V1.24.0^59^ was used to identify differentially expressed genes between two experimental conditions. Genes were considered differentially expressed if their expression levels differed by at least 2 folds with Benjamini–Hochberg adjusted p-values ≤ 0.05.

### Gene enrichment analyses

Differentially expressed genes were clustered according to their functional similarity by using integrative bioinformatics resources DAVID 6.8 (https://david.ncifcrf.gov/) under the default settings. Only clusters with enrichment scores of greater than 0.5 were considered.

### qRT-PCR analyses

Two μg of total RNAs were reverse transcribed by using random hexamers. Primers used for the target genes were generated commercially (Ward Medic; Supplementary Table S1). Two μg of cDNAs were used as the reaction templates. qRT-PCR analyses were conducted by using the thermo cycler (Rotor gene Q, Qiagen) with HotStarTaq DNA polymerase (Qiagen), 0.1 mM forward and reverse primers, 4 mM MgCl_2_, dNTPs (Promega), and SYBR green (Invitrogen) at various annealing temperatures. Melting curve analyses were performed to verify the specificity of the PCR products. All threshold signals obtained were analysed to be > 95% efficient. The optimal condition for each target gene was selected and the amplification products were analysed using the Q-Rex software version 1.0.1. Signals were normalised to the housekeeping *sigA* transcript. The results were presented as relative quantification by using the 2^-∆∆ct^ method.

### mCherry MTB generation

MKR superspreader and H37Rv were grown in 7H9 media until the optical density at 600 nm reached 0.8. Two molar glycine was added to each culture one day before harvesting. MTB cultures were washed several times in 10% glycerol and then electroporated as previously described^60^ with 4.6 µg of pCHERRY3 plasmid (Addgene, Plasmid# 24659)^61^. Transformants were selected with hygromycin (100 µg/mL; Invitrogen).

### Intracellular MTB survival assay

For intracellular survival assay, human monocytic THP-1 cells in 96-well black plates (7 × 10^4^ cell per well) were differentiated into macrophages with the treatment of 100 nM PMA (Sigma) for 24 h. The cells were then washed with complete media three times and rested in complete media for 24 h. Infection of macrophage cells with mCherry-expressing MKR superspreader or H37Rv (Fig. 5) and mycobacterial strains stained with Alexa-568^62^ (Fig. 7) were carried out for 30 min at MOI of 10 as described above. The infected cells were washed three times with complete media to remove uninternalised mycobacteria and were further incubated with complete media treated with different concentrations of V-59, aripiprazole, manidipine or ethoxzolamide (0, 3.125, 6.25, 12.5, and 25 μM) for 3 d. Cells were then fixed with 4% paraformaldehyde for 30 min, stained with Hoechst for 15 min, and subjected to high-content image analysis (Operetta, PerkinElmer) for counting the number of intracellular MTB per cell. Percent mycobacterial survival at each drug concentration was computed by dividing the observed number of intracellular MTB per cell by the average number from the non-treated control group multiplied by 100.

### Statistical analysis

Unless otherwise stated, all experiments were conducted at least three times and the data were pooled to compute the mean and the standard errors of mean. All data were analyzed by the Prism 5.0 software (GraphPad). *p* values corrected for multiple testing of less than 0.05 were considered to indicate statistical significance.

## Supplementary Information

Supplementary Dataset 1.

Supplementary Dataset 2.

Supplementary Dataset 3.

Supplementary Information.

## Data Availability

The data supporting the findings are available as supplementary materials.
