# Supplementary material for: Transcriptional response to the host cell environment of a multidrug-resistant Mycobacterium tuberculosis clonal outbreak Beijing strain reveals its pathogenic features
Source: Sci Rep. 2021 Feb 4;11:3199. doi: 10.1038/s41598-021-82905-x (PMC7862621; doi:10.1038/s41598-021-82905-x)
Supplement: Supplementary file 4 — Supplementary Information. [file 41598_2021_82905_MOESM4_ESM.pdf]

Supplementary Information

Transcriptional response to the host cell environment of a multidrug-resistant *Mycobacterium tuberculosis* clonal outbreak Beijing strain reveals its pathogenic features

Pakorn Aiewsakun[1,2], Pinidphon Prombutara[3,4], Tegar Adriansyah Putra Siregar[1], Thanida Laopanupong[1], Phongthon Kanjanasirirat[5], Tanawadee Khumpanied[5], Suparerk Borwornpinyo[5,6], Pirut Tong-Ngam[7], Alisa Tubsuwan[7], Prapaporn Srilohasin[8,9], Angkana Chaiprasert[8,9], Wuthiwat Ruangchai[2], Prasit Palittapongarnpim[1,2,10], Therdsak Prammananan[10], Brian C. VanderVen[11], and Marisa Ponpuak[1, 2*]

Supplementary materials and methods

CRISPRi gene expression knockdown and intracellular survival analysis

A 20-bp sequence immediately following the 5′-NGG-3′ was designed to target *kstR* and *espD* genes as previously described [1]. In brief, the respective target specific complementary oligonucleotides with *BsmBI* restriction sites immediately 5′ to the sgRNA, ONF and ONR (**Supplementary Table S2**), were synthesized, annealed, and subsequently cloned into the PLJR965 CRISPRi vector backbone at *BsmBI* restriction sites. The insertions of the sgRNA sequences were verified by direct sequencing using the 1834 primer (5′-TTCCTGTGAAGAGCCATTGATAATG-3′). The individual plasmid containing an sgRNA targeting gene was subsequently transformed as previously described [2] into the *M. tuberculosis* reference strain H37Rv or the MKR superspreader to reduce the expression of *kstR* and *espD* genes. Transformants were selected and maintained in 7H9 media containing kanamycin (30 µg/mL; Pacific BioLabs). Gene expression was knocked down by ATc induction (100 ng/mL; Sigma) for 4 d. Total RNA isolation and qRT-PCR analysis were conducted to determine the successful gene expression knockdown as described in the materials and methods.

For intracellular survival analysis, the gene expression knockdown was induced with ATc addition for 4 d as described above. The ATc-induced and ATc-uninduced mycobacteria were then washed with PBS and stained with Alexa-568 for 1 hr as previously described [3]. THP-1 cells were then infected with Alexa-568-labelled mycobacteria as described in the materials and methods, but in the complete media either supplemented or unsupplemented with ATc. The infected cells were washed three times with complete media to remove uninternalised mycobacteria and were further incubated with complete media supplemented or unsupplemented with ATc for 24 hr. Cells were then fixed with 4% paraformaldehyde for 30 min, stained with Hoechst for 15 min, and subjected to high-content image analysis (Operetta, PerkinElmer) for counting the number of intracellular MTB per cell. Percent mycobacterial survival of was then computed by dividing the number of intracellular MTB per cell to that of the average number of the ATc-uninduced control multiplied by 100.

Supplementary references

1 Rock, J. M. *et al.* Programmable transcriptional repression in mycobacteria using an orthogonal CRISPR interference platform. *Nat Microbiol* **2**, 16274; 10.1038/nmicrobiol.2016.274 (2017).

2 Goude, R., Roberts, D. M. & Parish, T. Electroporation of mycobacteria. *Methods Mol Biol* **1285**, 117-130 (2015).

3 Ponpuak, M., Delgado, M. A., Elmaoued, R. A. & Deretic, V. Monitoring autophagy during *Mycobacterium tuberculosis* infection. *Methods Enzymol* **452**, 345-361 (2009).

Supplementary Table S1. Primer sequences used for qRT-PCR in this study.

Gene name	Primer name	Sequence (5' – 3')	Annealing temperature (°C)
kstR	kstR_F	5'- TATGGTCGGCAAGCTCAACC -3'	55
	kstR_R	5'- GCGCGAACATACTGTCGATG -3'	
kshA	kshA_F	5'- AGCCGACATAAAACCAGAGA -3'	55
	kshA_R	5'- AAGTTCTTCTCTACCTCGGC -3'	
kshB	kshB_F	5'- CGGCAAAGTGAATATGGGAG -3'	52
	kshB_R	5'- CGAGATTGACAGGCCAAAAT -3'	
hsaA	hsaA_F	5'- AGATCAAGGACGTGTGGTAC -3'	55
	hsaA_R	5'- GGGGCACAAAGACATCCT -3'	
hsaC	hsaC_F	5'- CTGATGCTGTCGTTCTACATG -3'	52
	hsaC_R	5'- GATCCAGTCCCGATCGTC -3'	
fadE26	fadE26_F	5'- TGATTCAGTACGCCGACTAC -3'	52
	fadE26_R	5'- GATTAACACCGATATGCCACG -3'	
fadE28	fadE28_F	5'- CAAGTCGGTGATCTGGAGAC -3'	55
	fadE28_R	5'- CTGCGATGTCACCCAGTAC -3'	
cyp125	cyp125_F	5'- AGATTAAGAAGGGTCAGCGG -3'	55
	cyp125_R	5'- AAATGTGAACGGATCCTGGA -3'	
papA1	papA1_F	5'- GAAAGGATGACATGGCGGT -3'	55
	papA1_R	5'- ATCTACCGTCACATTCGCAA -3'	
espC	espC_F	5'- ATGTGTACTTGACTGCCCAC -3'	55
	espC_R	5'- CGCTATATATCTTCGCCGCA -3'	
espD	espD_F	5'- GTGGCCTGGATGAGTGAGTC -3'	55
	espD_R	5'- AAGGATGAAGGCGTACTGGG -3'	
esxB	esxB_F	5'- CCAAGAAGCAGCCAATAAGC -3'	55
	esxB_R	5'- CCTCGAGTATTGGACGCC -3'	
esxN	esxN_F	5'- ATCGTTCGTGATGTGTTGG -3'	57
	esxN_R	5'- CCAACTGGGTAATGAACTCC -3'	

Supplementary Table S2. List of oligonucleotides used for CRISPRi cloning.

Gene name	Oligo name	Sequence (5' – 3')
kstR	kstR_ONF	5'- GGGAGCCGACCATAAAGTTCAGCCG -3'
	kstR_ONR	5'- AAACCGGCTGAACTTTATGGTCGGC -3'
espD	espD_ONF	5'- GGGAGGTCGGCAATCACCAGGATC -3'
	espD_ONR	5'- AAACGATCCTGGTGATTGCCGACC -3'

Supplementary Dataset S1. Read mapping counts.

Supplementary Dataset S2. Differential gene expression analyses.

Supplementary Dataset S3. Functional gene enrichment analyses.

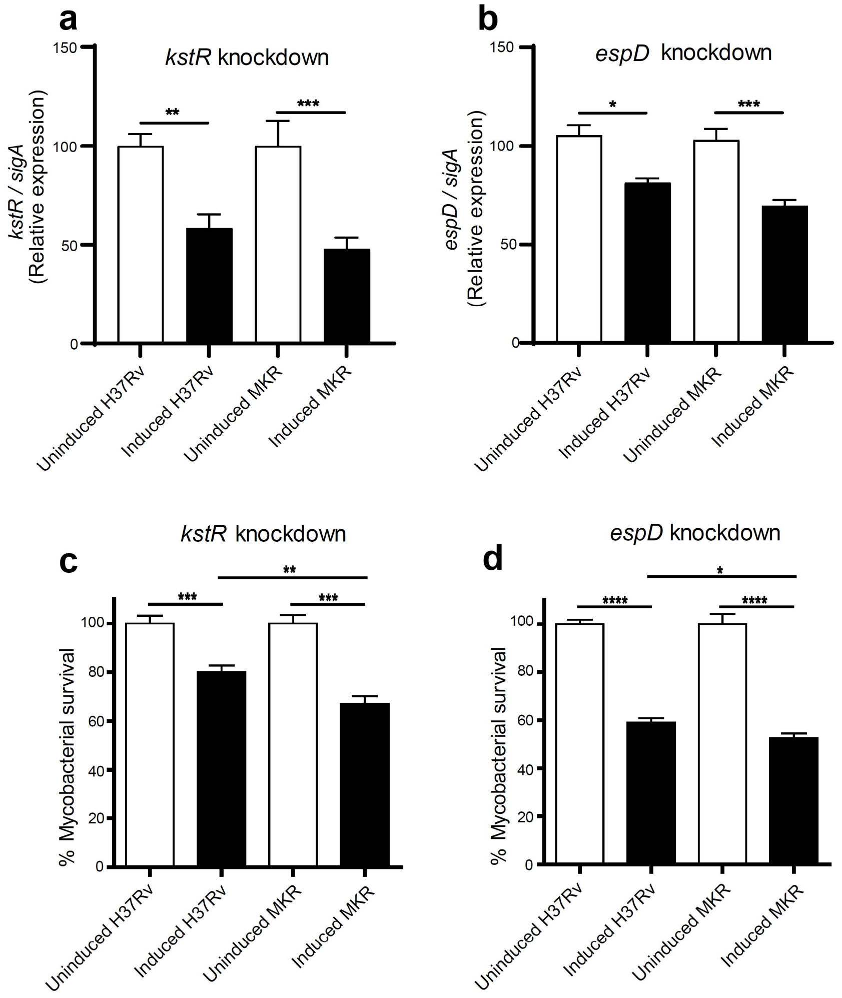

Supplementary Figure S1. qRT-PCR and mycobacterial survival analyses of KstR- and EspD-deficient mycobacteria. sgRNAs targeting *kstR* (**a**) and *espD* (**b**) were co-expressed with the dCas9 system in the H37Rv or MKR. Gene expression was knocked down by ATc induction for 4 d, and quantified by qRT-PCR. $2^{-\Delta\Delta ct}$ is used for normalisation and relative quantification. Data are presented as mean ± SEM of three independent experiments. One-way ANOVA with Tukey's multiple comparison test was used to determine pairs with significantly different gene expression levels (*:p < 0.05, **;p < 0.01, ***;p<0.001). *kstR*- or *espD*-CRISPRi targeting system expressing mycobacteria (**c** and **d**, respectively) were labelled with Alexa-568 and infected into THP-1 macrophages at MOI of 10 for 1 hr in the presence or absence of ATc. Cells were then washed three times with complete media to remove uninternalized mycobacteria, and were incubated further for 24 hr in the presence or absence of ATc. Following that, cells were fixed and processed for high-content image analysis to quantify the number of mycobacteria per host cell. Percent mycobacterial survival of each strain was calculated relative to that of the uninduced control set to 100%. Data are presented as mean ± SEM of three independent experiments. One-way ANOVA with Tukey's multiple comparison test was used to test if the intracellular survival levels were significantly different (*:p < 0.05; **: p < 0.01, ***;p<0.001, ****;p<0.0001).

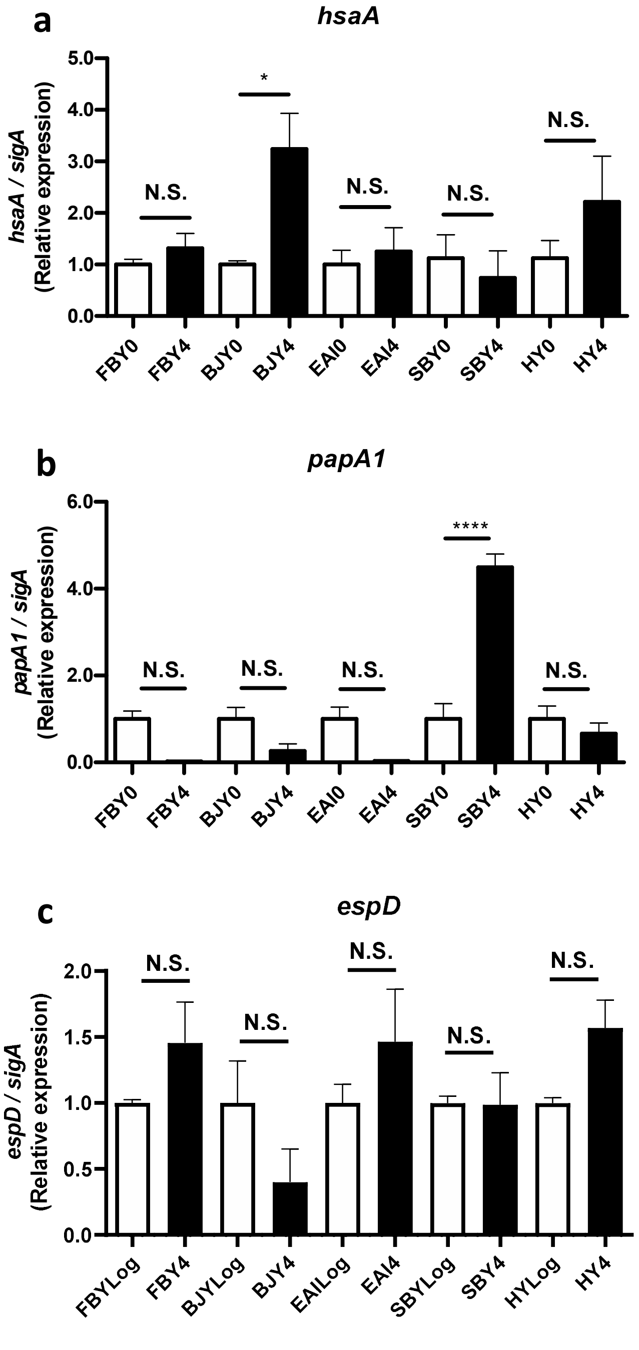

Supplementary Figure S2. qRT-PCR analyses of *hsaA*, *papA1*, *espD* mRNA expression in *M. tuberculosis* clinical isolates. (**a-c**) THP-1 macrophages were infected with *M. tuberculosis* clinical isolates as in Figure 4 and 6. The expression levels of *hsaA*, *papA1*, and *espD* were quantified by qRT-PCR. $2^{-\Delta\Delta ct}$ is used for normalisation and relative quantification. Data are presented as mean ± SEM of three independent experiments. One-way ANOVA with Tukey's multiple comparison test was used to test if the expression levels were significantly different (*:p < 0.05 and ****: p < 0.0001).
